# Phylogenetic and antimicrobial drug resistance analysis of *Vibrio cholerae* O1 isolates from Ghana

**DOI:** 10.1099/mgen.0.000668

**Published:** 2021-10-29

**Authors:** Japheth A. Opintan, Robert C. Will, George K. Kuma, Mary Osei, Amos Akumwena, Gifty Boateng, Godfred Owusu-Okyere, Lorreta Antwi, David Opare, Agila Kumari Pragasam, Karthick Vasudevan, Sunil Kumar Srivastava, Veeraraghavan Balaji, Mercy J. Newman, Gordon Dougan, Ankur Mutreja

**Affiliations:** ^1^​ Medical Microbiology Department, University of Ghana Medical School, Accra, Ghana; ^2^​ Department of Medicine, University of Cambridge, Cambridge, UK; ^3^​ Cambridge Institute of Therapeutic Immunology and Infectious Disease (CITIID), Department of Medicine, University of Cambridge, Cambridge, UK; ^4^​ Laboratory Department, Brong Ahafo Regional Hospital, Sunyani, Ghana; ^5^​ National Public Health Reference Laboratory, Accra, Ghana; ^6^​ Christian Medical College, Vellore, Tamil Nadu, India; ^7^​ Department of Biotechnology, School of Applied Sciences, REVA University, Bangalore, India; ^8^​ University of Delhi, Delhi, India; ^9^​ Translational Health Science and Technology Institute, Faridabad, India

**Keywords:** antibiotic resistance, cholera, outbreak, pandemic, phylogeny, West Africa

## Abstract

We investigated the evolution, phylogeny and antimicrobial resistance of *Vibrio cholerae* O1 isolates (VCO1) from Ghana. Outbreak and environmental sources of VCO1 were characterized, whole-genome sequenced and compared to globally available seventh pandemic (7P) strains of *V. cholerae* at SNP resolution. Final analyses included 636 isolates. Novel Ghanaian isolates clustered into three distinct clades (clades 1, 2 and 3) in wave 3 of the 7P lineage. The closest relatives of our novel Ghanaian isolates were from Benin, Cameroon, Togo, Niger and Nigeria. All novel Ghanaian isolates were multi-drug resistant. Environmental isolates clustered into clade 2, despite being isolated years later, showing the possibility of persistence and re-emergence of older clades. A lag phase of several years from estimated introduction to reported cases suggests pathogen persistence in the absence of reported cholera cases. These results highlight the importance of deeper surveillance for understanding transmission routes between bordering countries and planning tailored vaccination campaigns in an effort to eradicate cholera.

## Data Summary

All the new Ghanaian sequence data have been deposited in the National Center for Biotechnology Information (NCBI) GenBank Short Read Archive under BioProject accession number PRJEB20540. The full list of accession numbers is given within Table S1 (available with the online version of this article).

**Impact Statement**

While many studies have investigated *Vibrio cholerae* in the past, detailed molecular epidemiology work aimed at understanding the national dynamics of cholera in Ghana is rare. We collected 127 clinical and environmental O1 *V. cholerae* isolates and performed whole-genome sequencing, then reconstructed a global phylogeny to understand where our novel Ghanaian genomes fit. Our isolates clustered into three clades, each temporally distinct – suggesting numerous introduction events into the country with close relatives from West and Central African countries. All novel Ghanaian isolates were multi-drug resistant to a diverse range of antibiotics. beast analysis was used to date the important regional spread and estimated dates of cholera introduction(s) into Ghana. Evidence of persistence of clinical isolates in the environment was found, suggesting a continuous exchange between populations and their environments. Our findings provide an insight into the national and regional landscape of cholera disease in Ghana. Additionally, our work highlights the need for continued improved surveillance networks of transmission routes, especially focussing on neighbouring countries and trade routes in the region. These networks will be of paramount importance in the fight against cholera in this region, especially when planning vaccination campaigns.

## Introduction

Though an old disease, which has been eradicated in many industrialized countries, cholera is still persistent in many low- and middle-income countries. Cholera is a potentially lethal disease caused by the bacterium *Vibrio cholerae *, which is mainly transmitted through contaminated water and food [1]. The estimated incubation period of cholera can be between 12 h and 5 days [2]. Severe dehydration due to cholera can be fatal if fluid and electrolyte replacement is not promptly administered [3].

Over 200 serogroups of *V. cholerae* have been identified, but only O1 and O139 cause epidemics. Today, cholera outbreaks are almost exclusively caused by *V. cholerae* O1. Asymptomatic individuals and aquatic sources, including coastal areas, likely serve as the main reservoir for cholera transmission. Some studies have shown that climate changes may stimulate conditions that favour bacterial growth in coastal environments and promote the survival of epidemic *V. cholerae* isolates [4].

In some parts of Africa and Asia, cholera is endemic with high case fatality rates (CFRs). Africa alone reported about 3.7 million cases of cholera to the World Health Organization (WHO) between 1970 and 2012, with about 155 000 deaths [5]. Coastal West African cities, in particular, are affected regularly by outbreaks that often spread to inland cities [6]. Between 1979 and 2015, Ghana reported over 167 000 cases of cholera with 5851 deaths (mean CFR=4.05) to the WHO [5]. Within these decades, however, there were some periods without cholera. Between 1986 and 1989, there was a 4 year period of cholera absence. Approximately three decades later, another 1 year gap of reported incidence (2013) was observed in Ghana. During 2013, the National Public Health Reference Laboratory (NPHRL) in Ghana processed about 157 suspected cholera stool specimens, but none were laboratory confirmed to be *V. cholerae *. In the following year (2014), Ghana recorded one of its largest cholera outbreaks, affecting over 50 000 individuals. Though with a lower CFR (0.92%), 9 out of the then 10 regions of Ghana were affected in this single outbreak. Additionally, the 2014 outbreak continued into 2015 and was only declared over in the 45th epidemiological week of 2015 [7]. It appears that many susceptible individuals succumb to severe cholera following a major gap in disease incidence. For instance, after the first and second hiatus, reported cholera was 1.4- to 2.3-fold above the national average, respectively.

Globally, seven pandemic waves of cholera have been identified. These waves are distinct and, in past decades, significantly reduced the world’s population due to several million deaths [8–11]. The first pandemic started in 1817 and we are presently in the seventh (7P), which commenced in 1961 [12]. The *V. cholerae* O1 biotype El Tor (7PET) isolates known to be circulating in the current pandemic are genetically homogeneous [13, 14]. Three independent but temporally overlapping waves of 7PET are involved in the global transmission of cholera [10]. At least 12 (the last being T13) different introductions of 7PET have been detected in Africa, with one exported from Africa to Latin America [10, 14]. These analyses have been possible because of the availability of molecular tools such as whole-genome sequencing (WGS) and analysis [11]. WGS data can form the basis of a lineage typing tool, which is becoming less expensive and is now a proven way of tracking the evolution of cholera outbreaks across the world [10, 15, 16]. Surprisingly, to date, the interplay between epidemic cholera outbreaks from Ghana and the current global seventh pandemic lineage has only been minimally studied [15, 17, 18]. In the current study, we characterized outbreak and environmental *V. cholerae* O1 isolates from Ghana, to determine their temporal and phylogeographical relationship to the global pandemic lineages of cholera.

## Methods

### Study area

Ghana has four zonal Public Health Reference Laboratories (PHRLs), one each in the Greater Accra, Ashanti, Western and Northern regions. The Greater Accra zonal PHRL also serves as the NPHRL. Disease reporting and investigation in Ghana, including cholera outbreaks, are done using the Integrated Disease Surveillance and Response (IDSR) system [19]. During cholera outbreaks, disease control officers at local hospitals take rectal swabs of patients reporting with symptoms of cholera and complete IDSR forms. The swabs, which are kept in Cary Blair transport medium, are sent to zonal PHRLs for microbiological analyses within 48 h of collection. Data collated at the NPHRL are finally shared with the WHO, through the Disease Surveillance Department of the Ministry of Health, Ghana. For the environmental *V. cholerae* O1 isolates analysed in the current study, rivers running through major cholera-prone communities within Accra were sampled. Sampling was done once a week around mid-morning and afternoon. This sampling was done during the non-cholera outbreak period (January – April 2016), but these communities were previously affected by cholera outbreaks. In some parts of Accra, sanitation is poor and defecating directly into water bodies is the norm [20, 21].

### Human *V. cholerae* isolates

Randomly, we collected a set of archived clinical *V. cholerae* isolates from the NPHRL, a collection spanning 6 years (2010–2015). The isolates were pre-enriched in alkaline peptone water (APW) and sub-cultured onto thiosulfate citrate bile sucrose (TCBS) agar. Presumptive *V. cholerae* isolates were sub-cultured onto nutrient agar and further characterized using commercial polyvalent and monovalent antisera (Difco).

### Environmental *V. cholerae* isolates

Environmental *V. cholerae* were cultured from sampled rivers running through major cholera-prone communities within Accra during a non-outbreak period. Standard protocols were used for both the sampling and processing of sampled water [22, 23]. Surface water samples were aseptically collected into 1000 ml containers. Samples were kept in cold boxes and transported to our laboratories for microbiological investigations. For selective enrichment, a 10 ml thoroughly mixed water sample was added to an equal volume of double strength APW. For heavily polluted water, a 1 ml water sample was enriched in 10 ml single-strength APW. The enrichments were done in duplicate, and one set was incubated overnight at 37 °C and the other at 42 °C. Tubes showing signs of growth were sub-cultured onto TCBS agar plates and incubated overnight at 37 °C. Presumptive *V. cholerae* isolates were sub-cultured onto nutrient agar, and further characterized as above.

### Antimicrobial susceptibility testing

Antimicrobial-susceptibility testing was performed by the Kirby Bauer disc diffusion test and breakpoints were determined in accordance with Clinical and Laboratory Standards Institute guidelines [24] using whonet version 2020 [25]. The following antimicrobials (with their disc concentration in μg) were tested: tetracycline (30 μg) (TCY), cefotaxime (30 μg) (CTX), trimethoprim/sulfamethoxazole (1.25/23.75 μg) (SXT), chloramphenicol (30 μg) (CHL), erythromycin (15 μg) (ERY), nalidixic acid (5 μg) (NAL) and ciprofloxacin (5 μg) (CIP) (Mast Group). *Escherichia coli* ATCC 25922 was used as reference strain for quality-control purposes. Antibiotic-susceptibility data were stored and analysed with whonet [25].

### Bacterial DNA preparation

A pure culture of *V. cholerae* O1 on TCBS agar was inoculated into 3 ml trypticase soy broth and incubated overnight at 37 °C. Bacterial cells were pelleted, and genomic DNA was extracted using the Wizard genomic DNA purification kit (Promega), following the manufacturer’s recommendations.

### WGS and analysis

WGS for Ghanaian isolates was done on an Illumina HiSeq 2000 system at the Wellcome Sanger Institute, Hinxton, UK. Multiple sequencing libraries of 250 bp were created to perform 72 base paired-end sequencing of 96 separate libraries in each lane. Libraries were uniquely index tagged and used for assigning reads to the individual samples after sequencing.

To obtain a whole-genome alignment for all isolates in the study, the 72 base paired-end read data were combined with a global collection of publicly available genomes. These included representatives from previously published analyses, isolated in 55 countries across 59 years (including four previous genomes from Ghana, 1970 and 1971). These were mapped to N16961 (NCBI accession numbers AE003852 and AE003853) using smalt [26]. We used Gubbins (v2.4.1) to filter recombination and produce a SNP alignment [27]. We inferred a global phylogenetic tree based on all the SNPs recorded in our analysis using iq-tree (multicore version v1.6.10) and the inbuilt ModelFinder with 1000 pseudo-bootstraps [28, 29]. The details on novel Ghanaian isolates included for phylogenic analysis are shown in Table S1. FigTree and Interactive Tree of Life (iTOL v5.5.1) were used for visualization and ordering of nodes [30, 31]. BAPs clustering analysis (RhierBAPS v1.1.3) was used to investigate population structure [32].

In order to investigate the presence of a molecular clock (temporal signal) in the data, we employed root-to-tip genetic distances from a maximum-likelihood (ML) tree against sample collection dates using Tempest v 1.5.1 (http://tree.bio.ed.ac.uk). The recombination free alignment file was used as the input for the time-scaled phylogenetic analysis using the beast 1.10 package [33]. The Hasegawa, Kishino and Yano model (HKY) substitution with different demographic models (Bayesian skyline, exponential and constant) was investigated. Markov chain Monte Carlo (MCMC) runs of 100 million generations were carried out with sampling of 20 000 generations [34]. To determine the best-fitting coalescent model to describe changes in effective population size over time, log marginal likelihoods were calculated using path sampling and stepping stone sampling methods. Finally, Bayes factor was used to determine the best fit model with the formula [logBF=logPr(D|M1) – logPr(D|M2)]. The selected model was run in five independent chains for 100 million, with sampling of 10 000 generations. A burn-in of 20 % was discarded from each run and resulting log files were combined using LogCombiner 1.8.1 [35]. The convergence and mixing were manually inspected using Tracer.v.1.7 to ensure that all the parameters converged to an Estimated Sample Size of >200. A maximum clade credibility (MCC) tree was generated using Treeannotator v.1.8.2 [36]. The output was analysed using Tracer v1.7, with uncertainty in parameter estimates reflected as the 95 % highest probability density (HPD). The annotated phylogenetic tree was visualized using FigTree v.1.4.4 [37].

### Genotypic analysis of antimicrobial resistance (AMR) genes and *ctxB* variants

For the most comprehensive *in silico* AMR analysis, we used a combination of widely used tools to purify and analyse our reads. To ensure removal of any genome contamination, Kraken2 (v2.0.8), KrakenTools and Abacas (v1.3.1) were used to mask and remove reads that did not match *V. cholerae *. Both the Short Read Sequence Typing for Bacterial Pathogens (srst2 v0.2.0) tool and ARIBA (v2.14.6) were used to screen the genomic data for any AMR genes [38–42]. The SRST2 Antibiotic Resistance Gene-ANNOTation (arg-annot) database was used as the database of gene sequences [38, 43]. *In silico* PCR was used to investigate the presence of *ctxB* toxin variants [44]. Mutations in the *gyrA* and *parC* genes were identified using CholeraeFinder with manual identification of mutations sites [45].

## Results

### Three clades of Ghana-specific *V. cholerae*

**Fig. 1.**
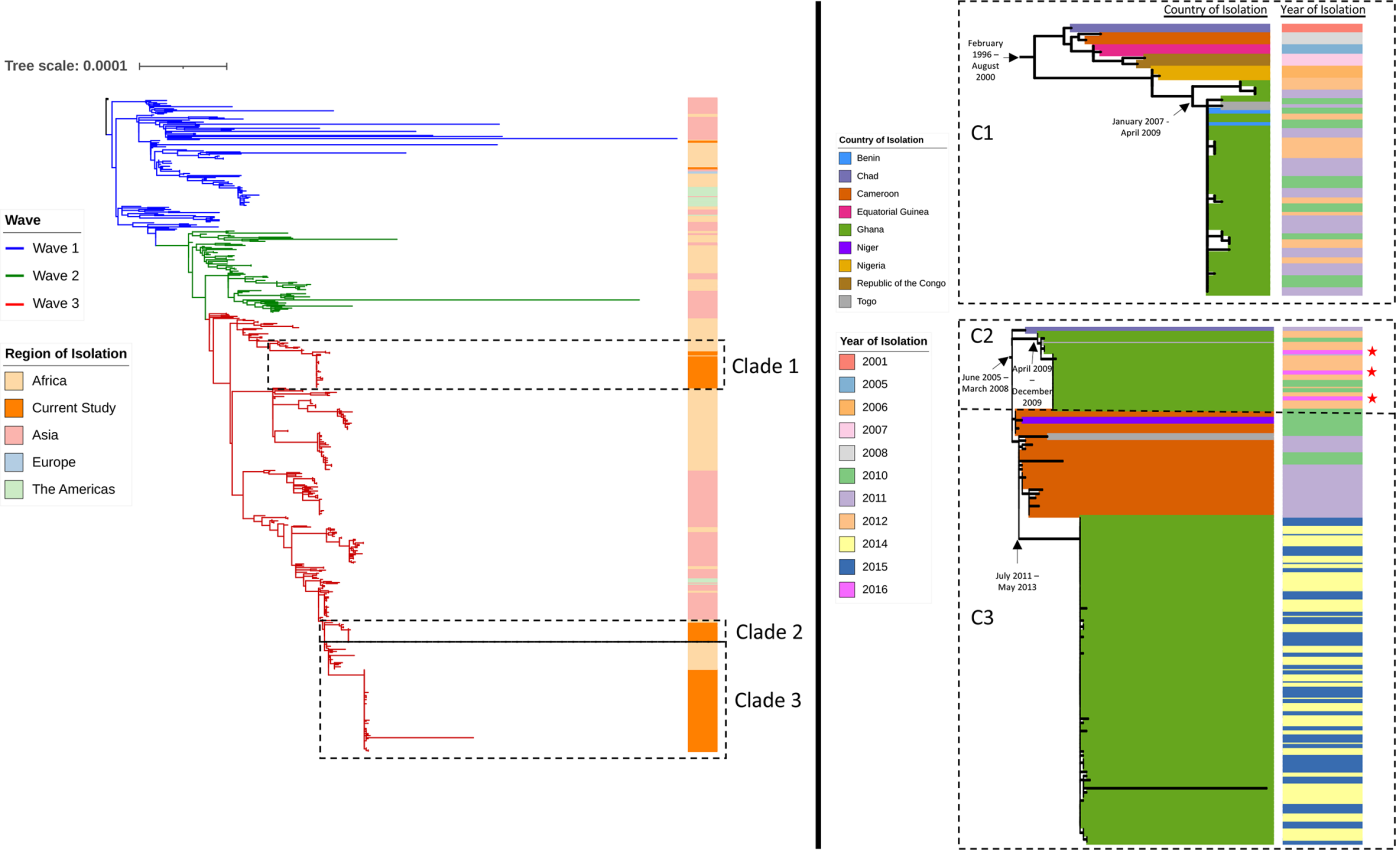
Inferred global phylogeny of 636 *V. cholerae* strains (novel Ghanaian isolates from this study, *n*=127, previously published sequence from Ghana, *n*=4, and global, *n*=505) SNPs (4676) showing waves 1, 2 and 3 of the current seventh pandemic lineages. *V. cholerae* strains are from Africa, Asia, Europe and the Americas. The tree scale indicates substitutions per site. The novel Ghanaian collection belongs to wave 3 of the current global seventh pandemic lineages, and clustered into three distinct clades (clades 1, 2 and 3). Ghanaian environmental *V. cholerae* O1 isolates included in our analysis clustered into clade 2. The country and year of isolation of the clades are indicated, with environmental isolates marked with a red star. Node dates calculated by beast are marked, showing the ancestor node of clades 1, 2 and 3, as well as each clade's estimated first introduction into Ghana.

A total of 135 novel *V. cholerae* O1 from Ghana (outbreak=127, environment=8) were confirmed to be O1 Ogawa serotypes by phenotypic characterization. Of these, 127 (outbreak=124, environment=3) were included in the final genome analysis. The rest were excluded from final analysis because they had <50 % coverage when mapped to the N16961 control strain and, thus, failed quality checks after checking for contamination. Sequenced genomes of the Ghanaian isolates included in the final analysis are publicly available at the European Nucleotide Archive with the accession codes listed in Table S1. Only three of eight environmental sources of *V. cholerae* O1 isolates had good coverage (>50 %) when mapped to the N16961 control strain. Fig. 1 shows an inferred global phylogeny of all 636 isolates analysed (Ghana, *n*=131, including 4 previously published sequences; global, *n*=505), based on 4676 SNPs. Global genomes were sourced from isolates collected across Africa, Asia, Europe and the Americas, the accession numbers of which can be found in Table S2. All novel Ghanaian isolates in this study fell into wave 3 of the current global seventh pandemic *V. cholerae* O1 lineages, while the four previously published sequences isolated in 1970 and 1971 mapped to wave 1. The novel Ghanaian isolates clustered into three distinct clades (1, 2 and 3), together with strains from other African countries. Whilst Ghanaian 2010–2012 isolates clustered into either clade 1 or 2, isolates from 2014 to 2015 clustered only into clade 3. The closest relatives of the Ghanaian collection were isolates from Cameroon, Togo, Benin, Niger and Nigeria (Fig. 1). Of note, all three Ghanaian environmental *V. cholerae* O1 isolates collected in 2016 clustered in clade 2 with 2010–2012 outbreak isolates. BAPs analysis identified seven clusters (Fig. S1), with clade 1 as part of cluster 7, and clades 2 and 3 as part of cluster 4. Clade 1 contains isolates previously reported as introduction event 9 (T9), while clade 3 contains isolates from introduction event 12 (T12), suggesting clade 2 is similarly from T12 based on the years of introduction [14].

### Introduction timings

The root-to-tip analysis revealed a strong correlation between the time of isolation and distance from the root, suggesting molecular clock-likeliness and robustness of the data. Bayesian skyline with uncorrelated relaxed clock was found to be the best fit model according to the Bayes factor analysis (Table S3). From the beast analysis, the evolution rate is estimated to be 6.376×10^−7^ substitutions per site per year. Fig. 1 shows the dated nodes as calculated by beast. Using 95 % confidence interval, we estimate that the most recent common ancestor of clade 1 strains existed between February 1996 and August 2000, and reached Ghana between January 2007 and April 2009. The shared ancestor of clades 2 and 3 is estimated to have diverged between June 2005 and March 2008. Clade 2 is estimated to have reached Ghana between April 2009 and December 2009, while clade 3 is estimated to have reached Ghana between July 2011 and May 2013.

### Stable antibiotic-resistance profiles that diversified over time

The phenotypic AMR profiles of the Ghanaian isolates are shown in Table S4. In general, all the isolates were multi-drug resistant, showing resistance to two or more classes of antibiotics. Isolates resistant to common and cheaper antimicrobials like trimethoprim/sulfamethoxazole and erythromycin were mostly observed between 2010 and 2012. Our isolate collection showed that until 2014, resistance to tetracycline, fluoroquinolones and cefotaxime were rare, becoming much more common in clade 3 (Table S1). However, no genes corresponding to these resistances were found in our *in silico* genome analysis.

**Fig. 2.**
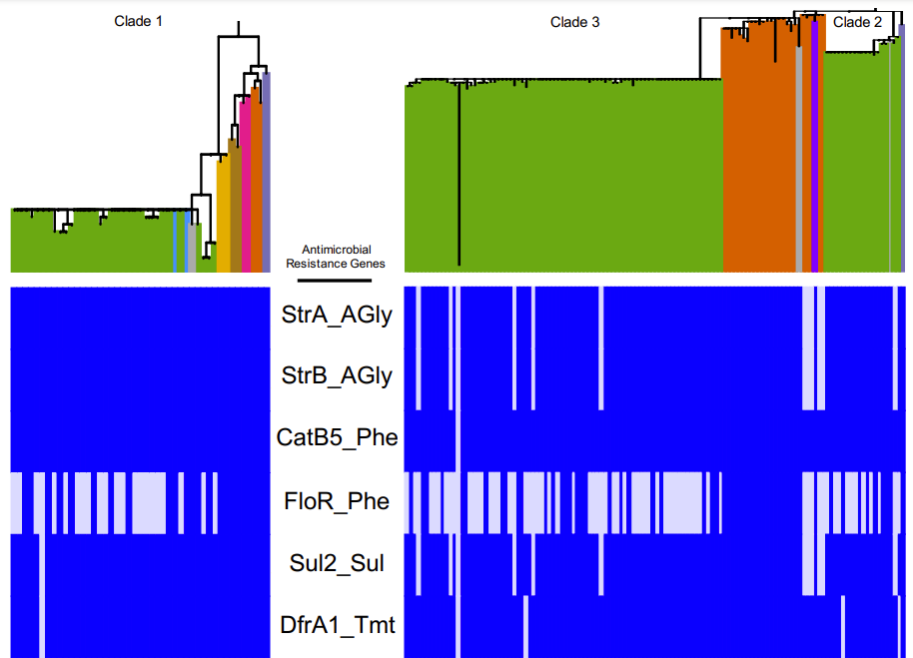
Antibiotic-associated genes identified across three distinct Ghanaian clades (clades 1, 2 and 3). Six genes known to confer resistance to streptomycin (*strA*, *strB*), florfenicol/chloramphenicol (*floR*, *catB5*), sulfonamide (*sul2*) and trimethoprim/sulfamethoxazole (*dfrA1* and *sul2*) were found in isolates spanning all clades, irrespective of year and country of isolation. Classes of antibiotics that each gene confers resistance to are shown next to the gene’s name: AGly, aminoglycosides; Phe, phenicols; Sul, sulfonamides; Tmt, trimethoprim.

Fig. 2 shows the presence or absence of antibiotic-associated genes across the three distinct Ghanaian clades, as identified by genomic analysis. Of note, six AMR genes (*strA*, *strB*, *catB5*, *floR*, *sul2* and *dfrA1*) were found across all three distinct clades, irrespective of their year and country of isolation. These genes are known to confer resistance to streptomycin (*strA*, *strB*), florfenicol/chloramphenicol (*floR*, *catB5*), sulfonamide (*sul2*) and trimethoprim/sulfamethoxazole (*dfrA1* and *sul2*) (Fig. 2). The three environmental *V. cholerae* isolates (strain numbers E20_16, E17_16 and E16_16) that were included in the final analysis carried similar antibiotic-associated genes (*strA*, *strB*, *catB5*, *floR*, *sul2* and *dfrA1*) (Table S1). Intriguingly, the three environmental isolates had similar genotypic gene content, yet phenotypically they expressed varying resistance profiles.

### Toxin variation and quinolone-resistance mutations are consistent with clades

Table S1 shows the *ctxB* variants determined using *in silico* PCR. All Ghanaian isolates had cholera toxin genes, with clade 1 isolates carrying only the *ctxB*_1 cholera toxin gene variant. In clades 2 and 3, isolates exclusively harboured the *ctxB*_7 variant. In clades 2 and 3, the S83I mutation in *gyrA* was and the S85L mutation in the *parC* gene were found in all of our novel Ghanaian isolates. In clade 1, however, no mutations were found in these genes among our novel Ghanaian isolates.

## Discussion

This whole-genome-sequence analysis of novel Ghanaian *V. cholerae* O1 isolates shows that they clustered into three separate and distinct clades. These novel *V. cholerae* O1 isolated between 2010 to 2016 mapped to wave 3 of the global seventh pandemic lineage of cholera. The four previous Ghanaian strains (1970 and 1971), which were isolated several decades earlier, mapped to wave 1. We provide evidence that the recent large cholera outbreak recorded in Ghana in 2014, which affected over 50 000 individuals [7], was introduced into the country between July 2011 and May 2013. From our inferred phylogeny, the closest relatives of the Ghanaian collection are strains from other West and Central African countries, including Cameroon, Togo, Benin, Niger and Nigeria. Our findings are corroborated by recent analysis of a collection of *V. cholerae* strains from the sub-region by Moore and colleagues (2018) [46]. Trade routes via road and boat may represent a major pathway by which cholera is imported from outbreaks in Nigeria, as both Benin and Nigeria have extensive trade links with Ghana [46]. The transmission of cholera is often along coastal routes to landlocked cites [6, 14, 18, 46]. Our analysis also suggests that there is a lag period of several years from the estimated introduction of each clade into Ghana to the appearance of cases caused by these strains. This clearly suggests that a temporary absence of reported cholera cases in these lag periods does not mean real absence of the pathogen itself, but instead a lack of any major outbreaks. Thus, these findings can aid in facilitating targeted control of future cholera outbreaks in the West African sub-region, recommending continual monitoring and checking importation routes should be a must for better understanding of transmission routes and implementing an efficient control strategy.

The environmental isolates from our study that were cultured from rivers running through major cholera-prone communities within Accra, during the non-outbreak period, clustered with 2010–2012 outbreak isolates in clade 2 of our inferred phylogeny. This observation suggests that a sub-set of 2010–2012 Ghanaian outbreak isolates may have persisted and remained in circulation, likely between asymptomatic carriers and faecal-contaminated environment. Intriguingly, however, these environmental isolates did not share a similar AMR profile with outbreak strains.

Our novel Ghanaian *V. cholerae* isolates belonging to wave 3 of the global seventh pandemic lineages were multi-drug resistant. Findings by Weill and colleagues in 2017 indicated that multi-drug resistant sub-lineages of 7P were introduced from Asia to Africa, and these replaced susceptible sub-lineages [14] . This is corroborated by several other studies from African countries including Ghana, where outbreak *V. cholerae* are beginning to develop resistance to effective antimicrobials like tetracycline and the fluoroquinolones [18, 47–51]. The observed mutations in *gyrA* and *parC* for all of the Ghanaian isolates in clades 2 and 3 are critical, as resistance to quinolones is an important evolutionary trait for sub-lineages of 7P [14]. Resistance-associated genes were identified by genome analysis in our study too, including streptomycin (*strA* and *strB*), chloramphenicol (*catB5* and *floR*), sulfonamides (*sul2*) and trimethoprim/sulfamethoxazole (*dfrA1*). Excluding *catB5*, the AMR genes identified here have previously been reported to be carried on integrative conjugative elements, including the ubiquitous ICE*Vch*Ind5 [52, 53].

In the current collection of 127 *V *. *cholerae* isolates, less than 15 % of our isolates showed resistance to tetracycline phenotypically. However, this has important clinical implications, as tetracycline is the drug of choice for managing cholera patients. In a similar cholera outbreak collection of 92 *V *. *cholerae* isolates within the same period (2011–2014), Eibach and colleagues (2016) did not identify any phenotypic resistance to chloramphenicol, gentamicin and tetracycline, especially in 2014 [17]. Eibach and colleagues, however, used 2015 Clinical and Laboratory Standard Institute breakpoints. In addition to the relatively higher number of isolates used in the current study, the 2020 version of whonet was used for determination of resistance. whonet is updated in real-time, has in-built quality checks and is recommended for breakpoint interpretation of susceptibility testing. Several other studies within Ghana [18, 50, 51] and elsewhere [54–56], within different time periods and using different breakpoints, have reported some resistance levels of *Vibrio* isolates to tetracycline. Due to the contamination removal from some of the Ghanaian *V. cholerae* genomes sequenced in this study, our *in silico* AMR analysis may have missed some rarer genes that are also shared between *V. cholerae* species and other closely related bacteria. This would also explain some of the discrepancies between our results on *in silico* and *in vivo* resistance determinants. Future analyses of *V. cholerae* in the region may provide further evidence of the development of resistance to tetracycline in Ghana and other neighbouring countries, in addition to providing further evidence on the widespread proliferation of SXT genes such as *floR*.

Our WGS analysis of a recent outbreak of cholera in Ghana shows that strains belonging to the same phylogenetic clades are circulating in several coastal West African countries at any given time. The extensive trade movement and a lack of understanding of transmission routes between these countries of the sub-region means a continuous monitoring programme would need to be put in place to efficiently block the cross-border movement of pandemic *V. cholerae *. Only once this is achieved will eradication by means of targeted vaccination programmes become thinkable.

## Supplementary Data

Supplementary material 2
